# Supplementary material for: Does the Timing of Response Impact the Outcome of Relapsed/Refractory Acute Myeloid Leukemia Treated with Venetoclax in Combination with Hypomethylating Agents? A Proof of Concept from a Monocentric Observational Study
Source: J Clin Med. 2025 Aug 7;14(15):5586. doi: 10.3390/jcm14155586 (PMC12347298; doi:10.3390/jcm14155586)
Supplement: Supplementary file 1 [file jcm-14-05586-s001.zip › jcm-3776507-supplementary.pdf]

Supplementary Table S1. Detailed TTR analysis

Parameter	*Subgroup*	*N*	*Median TTR (months)*	*95% CI*	*p-value*
Age	<50	7	15.9	6.0 -25.8	0.931
	≥50	12	15.9	10.8 -21.0	
Gender	Female	10	15.9	1.4 -30.4	0.362
	Male	9	11.9	10.0 – 13.7	
Prior therapy	HMA (AZA or DEC)	11	16.0	6.8 -29.3	0.209
	CHT and AZA	6	11.9	2.3 -16.3	
	CHT	2	37.3	Not evaluable	
R/R status	REL	13	15.9	9.1 -22.7	0.822
	REF	6	16.0	9.2 -22.8	
REL timing	Early	8	11.2	1.4 – 21.0	0.087
	Late	5	37.3	Not evaluable	
HMA backbone	Azacitidine	18	16.0	8.5 -23.5	0.747
	Decitabine	1	15.9	Not evaluable	
NPM1	Wild-type	16	15.9	10.8 - 21	0.447
	Mutated	3	4.2	1.2 -7.1	
Complex karyotype	No	15	15.9	10.8 -21.0	1.0
	Yes	4	11.2	1.3 – 21.2	
Late responder	No	12	16.0	5.0 -27	0.837
	Yes	7	15.9	11.5 – 20.3	
Allogeneic HSCT	No	10	15.9	10.4 -21.4	0.862
	Yes	9	11.2	Not evaluable	

Abbreviations*: ECOG PS: Eastern Cooperative Oncology Group Performance Status; HMA: Hypomethylating agents (Azacitidine [AZA] or Decitabine [DEC]); CHT: Chemotherapy; REL: Relapsed disease; REF: Refractory disease; HSCT: Hematopoietic stem cell transplantation*

Supplementary Table S2. Detailed RFS analysis

Parameter	*Subgroup*	*N*	*Median RFS (months)*	*95% CI*	*p-value*
Age	<50	7	16.0	6.1–25.9	0.426
	≥50	12	11.2	6.4–16.1	
Gender	Female	10	11.2	1.7–20.7	0.514
	Male	9	11.9	10.0–13.7	
Prior therapy	HMA (AZA or DEC)	11	9.0	1.9–16.1	0.800
	CHT and AZA	2	37.3	37.3–37.3	
	CHT	6	11.9	9.0–14.7	
R/R status	REL	13	13.6	9.6–17.6	0.655
	REF	6	9.0	0.4–17.6	
REL timing	Early	8	11.2	1.4–21.0	0.174
	Late	5	37.3	Not evaluable	
Line of therapy	2nd	17	11.9	6.0–17.8	0.546
	≥3rd	2	11.2	Not evaluable	
HMA backbone	AZA	18	11.2	9.8–12.7	0.991
	DEC	1	15.9	Not evaluable	
NPM1	Wild-type	16	11.9	7.5–16.2	0.657
	Mutated	3	4.2	1.2–7.1	
Complex karyotype	No	15	13.6	8.4–18.8	0.719
	Yes	4	4.7	0.0–11.3	
Late responders	No	12	11.2	0.0–26.5	0.547
	Yes	7	11.9	10.2–13.5	
Allogeneic HSCT	No	10	15.9	10.4–21.4	0.263
	Yes	9	11.2	4.8–17.5	

Abbreviations: *ECOG PS: Eastern Cooperative Oncology Group Performance Status; HMA: Hypomethylating agents (Azacitidine [AZA] or Decitabine [DEC]); CHT: Chemotherapy; REL: Relapsed disease; REF: Refractory disease; HSCT: Hematopoietic stem cell transplantation*

Supplementary Table S3. Detailed OS analysis

Parameter	*Subgroup*	*N*	*Median OS (months)*	*95% CI*	*p-value*
Age	< 50	7	17.9	10.3–25.4	0.067
	≥ 50	26	8.1	6.6–11.3	
Gender	Female	18	7.1	0.0–14.2	0.578
	Male	15	13.6	4.7–22.5	
ECOG PS	0–2	28	11.2	2.7–19.6	0.065
	3–4	5	7.1	0.6–13.6	
Prior therapy	HMA (AZA or DEC)	14	8.4	2.9–14.0	0.759
	CHT and AZA	7	14.5	0.0–29.8	
	CHT	12	8.3	0.0–17.4	
R/R status	REL	26	8.4	3.7–13.0	0.956
	REF	7	9.0	0.0–18.2	
REL timing	Early	17	8.2	6.5–10.0	0.176
	Late	9	14.5	6.4–22.7	
Line of therapy	2nd	26	8.4	7.1–9.6	0.753
	≥3rd	7	11.2	5.4–21.7	
HMA backbone	AZA	28	11.2	4.2–18.1	0.183
	DEC	5	8.2	1.4–15.1	
NPM1	Wild-type	26	8.4	4.7–12.0	0.759
	Mutated	7	14.2	0.0–36.6	
Complex karyotype	No	28	9.0	2.5–15.5	0.962
	Yes	5	5.4	4.6–6.2	
Response	**Responder**	**19**	**15.9**	**12.6–18.6**	**<0.001**
	**Failure**	**14**	**5.0**	**2.7–7.4**	
Late responders	No	12	17.9	9.0–26.8	0.437
	Yes	7	15.6	10.5–20.7	
Allogeneic HSCT	No	24	8.1	4.8–11.3	0.454
	Yes	9	13.6	7.5–19.7	

Abbreviations*: ECOG PS: Eastern Cooperative Oncology Group Performance Status; HMA: Hypomethylating agents (Azacitidine [AZA] or Decitabine [DEC]); CT: Chemotherapy; REL: Relapsed disease; REF: Refractory disease; HSCT: Hematopoietic stem cell transplantation*
